# Environmental Contamination in Nigeria

**DOI:** 10.5696/2156-9614-7-13.1

**Published:** 2017-03-29

**Authors:** Jack Caravanos

**Figure:**
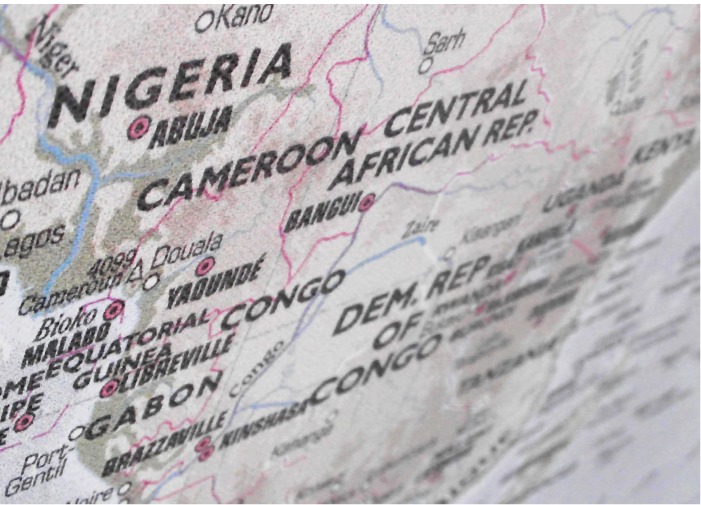


Nigeria has come a long way. In addition to being the most populous country in Africa, and the 7th largest worldwide, its economy is larger than that of South Africa. With over 128 universities, it is considered to have the strongest educational system in West Africa, focusing on science and technology.

The country is the 12th largest producer of crude oil and the contamination of the Niger Delta is regrettably infamous. Other environmental health issues such as untreated sewage in Lagos, open solid waste dumpsites in Abjua, lead poisoning in the northern state of Zamfara, and large scale deforestation have all contributed to the need for enhancing research and scholarship.

Therefore, it is no surprise that *JHP* and other journals have seen an increase in manuscript submissions from Nigerian universities and ministries. As such, this issue of *JHP* is a compilation of research articles focusing on the environmental health of Nigeria.

The article by Ojo, Onayade, Akinyemi, and Adesanmi describes and quantifies the various occupational hazards associated with commercial spray painting operations in Nigeria.

Regrettably, workplace hazards due to use of solvents, metals and gases are all too prevalent in this rapidly developing country.

Oguguah and Ikegwu study the concentrations of heavy metals in fish—an important part of the Nigerian diet.

Etim from the University of Ibadan presents important data on arsenic, antimony and selenium in the shallow tube wells of southwestern Nigeria. The complicated chemistry of these metals highlights the challenge in assessing toxicity and ultimate public health impacts.

With regard to soil contamination, Adeyi and Babalola present findings on the ongoing seemingly ubiquitous lead and cadmium contamination of urban areas. While the values observed were below levels observed at used lead acid battery recycling sites, the fact remains that historical lead in soil contamination is ever present worldwide. Oyeyiola, Adeosun and Fabunmi report on a low cost technique to reduce the bioavailability of metals in contaminated soils.

Finally, Ipeaiyeda and Adegboyega's air pollution study confirms numerous other observations that levels of SO_2_, NOx and other criteria air pollutants further exacerbate the particulate pollution reported in recent press articles.

We congratulate the hard working scholars of southern Nigeria for helping to bring attention to environmental and occupational health issues in this vital country and hope such efforts might spread to other neighboring countries.

